# Ca^2+^ Signaling and ATP Production in Pancreatic Cancer

**DOI:** 10.1093/function/zqad067

**Published:** 2023-12-08

**Authors:** Julia V Gerasimenko, Oleg V Gerasimenko

**Affiliations:** Cardiff School of Biosciences, Cardiff University, Museum Avenue, Cardiff, CF10 3AX, UK; Cardiff School of Biosciences, Cardiff University, Museum Avenue, Cardiff, CF10 3AX, UK

**Keywords:** pancreatic cancer, pancreatic adenocarcinoma, calcium signaling in pancreatic cancer, ATP production

## A Perspective on “Driver Mutations of Pancreatic Cancer Affect Ca^2+^ Signaling and ATP Production”

Pancreatic cancer is a growing burden on public health in modern society representing a diagnostic and therapeutic challenge.^1^ Pancreatic adenocarcinoma (PDAC) is the predominant type of pancreatic exocrine neoplastic malignancy and the fourth most common cause of cancer-associated mortality worldwide, with the prediction to become the second most common cause of cancer death in a few years. It is also the deadliest type of malignancy, having the lowest 5-year survival rate of less than 8% with limited options for an effective treatment at the time of diagnosis.^2^,^3^ Although the progression from benign pancreatic neoplasia to malignant PDAC can take more than 20 years, in the absence of specific symptoms and with a lack of diagnostic markers, the early stages of the disease continue to be undetectable.^4^

It has been demonstrated that the risk of PDAC development depends on the presence of specific gene mutations such as *KRAS, BRCA1/2, TP53, ATM, MLH1*, and *CDKN2A*.^4^ However, additional risk factors include aging, obesity, type 2 diabetes, smoking, and inflammatory diseases of the pancreas, namely, acute and chronic pancreatitis.^4^,^5^

To this day, a great deal of uncertainty remains about the cell origin of PDAC.^4^ In spite of the ductal phenotype of PDAC and the associated terminology for this type of pancreatic cancer, growing evidence suggests that both pancreatic exocrine acinar and ductal cells can undergo neoplastic transformation leading to tumor development. However, it has been shown that the capacity of such transformation in different cell populations, either acinar or ductal, could be variable due to cellular heterogeneity within pancreatic neoplasms. Pancreatic acinar cells experience a higher level of plasticity due to the process of metaplasia that involves acinar-to-ductal reprogramming in response to acute injury of the pancreas. Therefore, pancreatic acinar cells are estimated to be ∼100 fold more likely to cause PDAC in the presence of prolonged exposure to stress and proto-oncogenic hits, prioritizing activation of *KRAS* mutations.^6^

It has been well established that calcium (Ca^2+^) signaling is involved in many pathological processes, including cancer by creating a new balance and communication within the tumor microenvironment, supporting tumor cell survival, proliferation, and migration.^7^ Therefore, investigation of pancreatic cancer-associated Ca^2+^ signaling pathways should be prioritized in the identification of specific markers and therapeutic targets against pancreatic carcinogenesis and invasiveness. A recent study by Stopa et al. has investigated Ca^2+^ signals and ATP levels in freshly isolated pancreatic acinar cells from Kras^G12D/+^, Trp53^R172H/+^, and Pdx-1-Cre (KPC) mice as a genetic model of PDAC.^8^ Their results highlight the ability of KPC pancreatic acinar cells to generate Ca^2+^ oscillations in response to the physiologically relevant concentration of acetylcholine (ACh). This secretagogue is well known to initiate Ca^2+^ release from internal stores through inositol trisphosphate receptors (IP_3_Rs) triggering secretion of zymogens from pancreatic acinar cells to aid food digestion. Stopa et al.^8^ have identified a significant increase in the amplitude of such Ca^2+^ oscillations in KPC cells as compared to control-Cre acinar cells; however, Ca^2+^ signals in response to a supramaximal concentration of ACh were notably smaller. These results could indicate that either the endoplasmic reticulum Ca^2+^ replenishment or Ca^2+^ extrusion or both are affected in KPC cells. It has been shown previously that some of the players in the Ca^2+^ signaling network, namely, intracellular Ca^2+^ releasing channels such as IP_3_Rs as well as store-operated Orai1/Ca^2+^ release activated Ca^2+^ (Orai1/CRAC) channels have been implicated in metastasis formation. The upregulation of plasma membrane Ca^2+^ ATPase (PMCA) isoform 1 has been identified and in general, PMCA was found to be responsible for the elevated level of Ca^2+^ efflux, cell migration, and resistance of cancer cells to apoptosis.^7^ Stopa et al. have demonstrated that the PMCA expression was not significantly different in KPC acinar cells as compared to control. Cancerous cells may exhibit specific preferences for Ca^2+^ efflux by expressing different PMCA isoforms, which leaves another open question about the expression of the sodium calcium exchanger in cancer.^7^ Interestingly, in this paper,^8^ the rates of Ca^2+^ extrusion in KPC acinar cells were significantly slower than in control but demonstrated a specific biphasic pattern. Potentially, this could lead to Ca^2+^ overload and apoptosis initiation, which is typically avoided in cancer cells. Therefore, the efficiency and adaptation of the above Ca^2+^ signaling mechanisms in neoplastic cells, which are crucial for the tumor establishment and survival, should be further investigated.

At the same time, due to high metabolic demands, cancer cells rewire cellular energy metabolism to fuel rapid cell growth, division, survival, and migration.^9^ To increase ATP production in the face of a hypoxic tumor microenvironment, cancer cells enhance aerobic glycolysis (the Warburg effect) and glucose uptake. However, recent studies have demonstrated that increased mitochondrial ATP generation is equally important for pancreatic cancer progression.^10^ Therefore, the result from Stopa et al.^8^ showing a reduction of ATP in KPC acinar cells is rather surprising; however, this could be explained by the stochastic expression of Cre recombinase in the pancreas.

It has been shown previously that the increased proton leak from mitochondria in Kras^G12D^-expressing cancer cells results in the production of reactive oxygen species (ROS).^10^ However, the ROS production in cancer cells should be maintained below a certain threshold to be able to promote acinar cell dedifferentiation to duct-like progenitors whilst at the same time restricting the induction of apoptosis. In the study by Stopa et al.,^8^ it appears that the mitochondrial proton leak was not altered in KPC acinar cells; nevertheless, it would be advantageous to directly measure ROS production as well as apoptosis in these cells. This may of course turn out to be a difficult project due to the heterogeneity of cells in the samples including normal, pre-malignant, and malignant acinar cells.

In conclusion, the pioneering research by Stopa et al.^8^ has highlighted the importance of Ca^2+^ signaling in PDAC with a unique approach and intriguing results on the aberrant Ca^2+^ and metabolic “signatures” in cancer cells that are clearly at the heart of pancreatic cancer progression. These data could provide important insights into cancer development and discover new therapeutic targets for the deadliest human cancer.
